# Comprehensive Insights into Biological Roles of Rosmarinic Acid: Implications in Diabetes, Cancer and Neurodegenerative Diseases

**DOI:** 10.3390/nu15194297

**Published:** 2023-10-09

**Authors:** Md. Khabeer Azhar, Saleha Anwar, Gulam Mustafa Hasan, Anas Shamsi, Asimul Islam, Suhel Parvez, Md. Imtaiyaz Hassan

**Affiliations:** 1 Department of Biosciences, Jamia Millia Islamia, Jamia Nagar, New Delhi 110025, India; khabeer.azhar146@gmail.com; 2 Department of Toxicology, School of Chemical & Life Sciences, Jamia Hamdard, New Delhi 110062, India; email2saleha@gmail.com; 3 Department of Basic Medical Science, College of Medicine, Prince Sattam Bin Abdulaziz University, P.O. Box 173, Al-Kharj 11942, Saudi Arabia; mgulam@gmail.com; 4 Centre of Medical and Bio-Allied Health Sciences Research, Ajman University, Ajman 364, United Arab Emirates; 5 Centre for Interdisciplinary Research in Basic Sciences, Jamia Millia Islamia, Jamia Nagar, New Delhi 110025, India; aislam@jmi.ac.in (A.I.); mihassan@jmi.ac.in (M.I.H.)

**Keywords:** anticancer therapeutics, phytochemicals, neurodegenerative diseases, rosmarinic acid, antioxidant, anti-inflammatory, drug discovery

## Abstract

Phytochemicals are abundantly occurring natural compounds extracted from plant sources. Rosmarinic acid (RA) is an abundant phytochemical of *Lamiaceae* species with various therapeutic implications for human health. In recent years, natural compounds have gained significant attention as adjuvant and complementary therapies to existing medications for various diseases. RA has gained popularity due to its anti-inflammatory and antioxidant properties and its roles in various life-threatening conditions, such as cancer, neurodegeneration, diabetes, etc. The present review aims to offer a comprehensive insight into the multifaceted therapeutic properties of RA, including its potential as an anticancer agent, neuroprotective effects, and antidiabetic potential. Based on the available evidences, RA could be considered a potential dietary component for treating various diseases, including cancer, diabetes and neurodegenerative disorders.

## 1. Introduction

Rosmarinic acid (RA) was first reported in the Lamiaceae family plants even before its chemical structure was elucidated [1]. Italian chemists successfully isolated pure RA from rosemary (*Rosmarinus officinalis*, *Lamiaceae*) in 1958, and chemical analysis revealed its structure and composition. RA is a derivative of the ester of caffeic acid (3,4-di-hydroxycinnamic acid) and 3,4-dihydroxyphenyllactic acid (DHPL) [2]. Researchers found that compounds extracted from aromatic herbs such as rosemary (*Rosmarinus officinalis* L.), sage (*Salvia officinalis* L.), thyme (*Thymus vulgaris* L.), lavender (*Lavendula angustifolia* Mill.), etc., possess high anti-inflammatory, antioxidant, antidepression, and antiallergy properties [3,4,5,6]. Polyphenols are known for their antioxidant properties and have been studied for their potential health benefits; RA is a vital member of this class, having numerous health benefits [7,8].

RA is present in diverse range of plants from *Boraginaceae, Lamiaceae* (restricted to sub-family Nepetoideae), Blechnaceae (family of ferns), hornworts (lower Plants), Zosteraceae (monocotyledonous plants like the sea grass), Potamogetonaceae, and Cannaceae. RA is categorized as a polyphenolic secondary metabolite and exhibits a wide range of pharmacological attributes. These include its ability to prevent the oxidation of low-density lipoprotein (LDL), inhibit murine cell proliferation, act as a cyclooxygenase inhibitor, and exert antiallergic effects. RA exhibits anti-inflammatory, antioxidant, astringent, antimutagenic, antibacterial, and antiviral properties [1,9,10,11]. Thus, in lieu of its broad therapeutic potential, RA has captivated the attention of researchers.

In a diabetic rat model treated with RA, there is an anti-inflammatory activity reported. This activity is achieved through the inhibition of the expression of various proinflammatory factors, including interleukin-6 (IL-6), interleukin-1β (IL-1β), tumour necrosis factor-alpha (TNF-α), and endothelin-converting enzyme-1 (ECE-1) [12]. RA deciphers its anti-inflammatory and antioxidant properties by inhibiting NF-κB activity and reducing the production of prostaglandin E2 (PGE2), nitric oxide (NO), and cyclooxygenase-2 (COX-2) in RAW 264.7 cells. This mechanism helps to prevent inflammation and oxidative stress [13]. RA inhibits cytotoxicity in tumour patients by maintaining the mitochondrial membrane potential [14]. In U937 cells, treatment with RA demonstrated anticancer effects mediated by TNF-α. These effects were achieved by inhibiting TNF-α-mediated generation of reactive oxygen species (ROS), apoptosis, and activation of NF-κB [15].

RA exerts antitumour effects in mice by suppressing the expression of mRNA for various proinflammatory and protumour factors, including Intercellular Adhesion Molecule 1 (ICAM-1), Vascular Cell Adhesion Molecule 1 (VCAM-1), Prostaglandin E2 (PGE2), Leukotriene B4 (LTB4), and COX-2. This suppression contributes to the anti-tumour effects of RA [16,17].

RA possesses antiallergic properties and can alleviate allergic inflammatory reactions, such as allergic rhinitis and rhinoconjunctivitis. It achieves this by inhibiting the expression of proinflammatory cytokines, including IL-1β, IL-6, and TNF-α, as well as suppressing COX-2 protein expression and caspase-1 activity in nasal mucosa tissue [18]. This review aims to assess the current updates on roles of RA in diabetes, cancer, and neurodegenerative disorders, providing valuable insights into its potential as a therapeutic agent. A better understanding the implications of RA in these diseases may pave the way for the development of effective therapeutic strategies or dietary interventions.

## 2. Chemical Synthesis of RA

**Figure 1:**
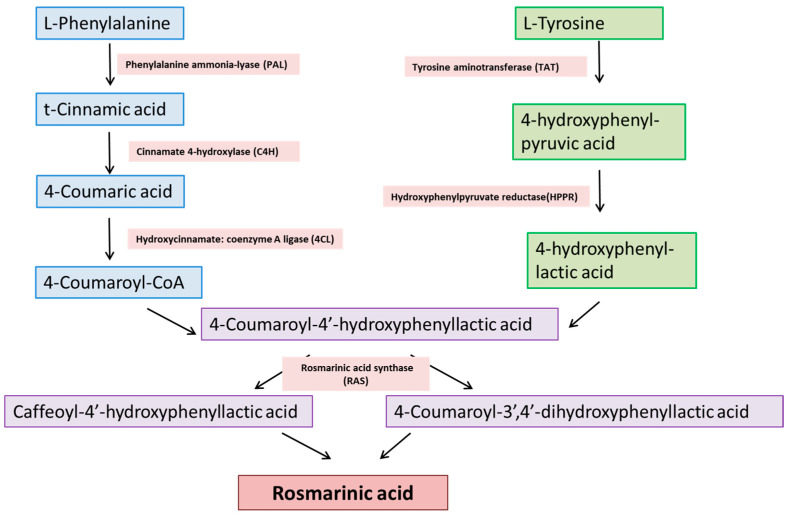
Biosynthetic pathway of RA synthesis.

The phenylpropanoid pathway is the common pathway for the biosynthesis of polyphenolic compounds such as flavonoids, specifically RA. Its biosynthesis starts with the deamination of l-phenylalanine to t-cinnamic acid by phenylalanine ammonia-lyase (PAL), and then hydroxylation of t-cinnamic acid at the fourth position by cinnamic acid 4-hydroxylase that leads to the formation 4-coumaric acid. The entire pathway for the biosynthesis is shown in Figure 1.

## 3. Therapeutic Implications of RA

RA is a naturally occurring polyphenolic compound found in various plants. It belongs to the class of compounds known as phenolic acids, and is a derivative of caffeic acid and related to other bioactive compounds such as flavonoids. Polyphenolic compounds are highly effective against numerous diseases, such as peptic ulcers, carcinogenesis, ischaemic heart disease, tumour cell proliferation, hyperglycaemia, atherosclerosis, apoptosis, etc. Polyphenols demonstrate potent capabilities as anti-inflammatory, antiallergy, antioxidant, antimicrobial, antiviral, and anticancer agents [19,20]. RA is a functional component of numerous medicinal plants. Various studies have demonstrated a wide range of biological activities associated with RA [21].

**Figure 2:**
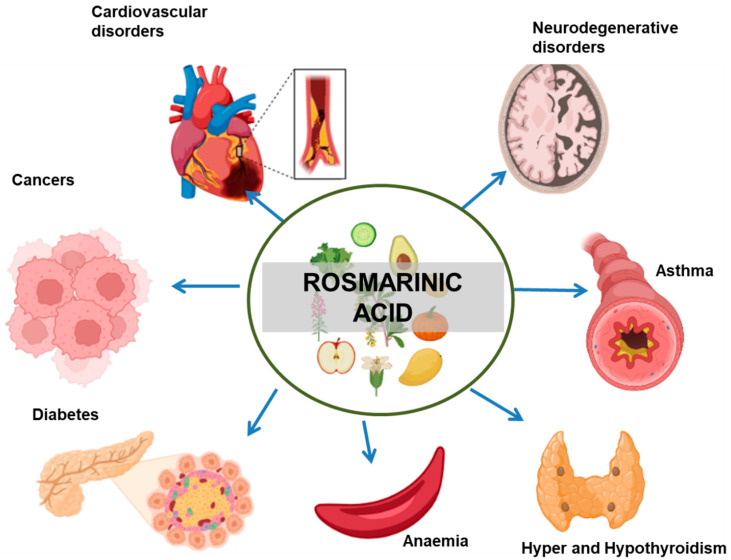
Therapeutic implications of RA in different diseases. The arrows indicate the negative effect of RA in the progression of the mentioned medical conditions.

RA shows a wide range of biological activities. It used as an additive, flavouring agent, and supplement. Figure 2 gives an overview of different biological activities associated with RA. Table 1 shows the role of RA in different diseases.

### 3.1. Role of RA in Diabetes Mellitus

Diabetes mellitus (DM) comprises a group of metabolic disorders characterized by chronic hyperglycaemia, which is the presence of elevated sugar levels in the bloodstream. This condition can result from either irregular insulin secretion, inappropriate insulin action, or a combination of both factors. DM has emerged as a substantial and escalating global concern within the public health sector. Currently, the available drugs for diabetes are efficacious to a limit and have some safety issues like hypoglycaemia, weight gain, and gastrointestinal side effects. RA has shown potential therapeutic effects in the context of DM treatment through various ways. Owing to RA’s antioxidant and anti-inflammatory activities, significant researchers have focused on investigating the potential of RA in DM. Various studies suggest that the extract of *Rosmarinus officinalis*, along with its phenolic constituents, particularly RA, carnosic acid, and carnosol, regulate glucose metabolism, lipid metabolism, anti-inflammation, and anti-oxidation, which ratchet up the therapeutic value against DM [46].

DM treatment encompasses more than managing appropriate blood sugar levels; it also involves addressing insulin resistance, improving insulin sensitivity, and ensuring the proper function of beta cells [47]. A research study explored how RA influences the regulation of glucose levels and insulin in two animal models mimicking diabetes: one induced to resemble type 1 diabetes using streptozocin (STZ) in rats, and the other induced to resemble type 2 diabetes through a high-fat diet (HFD) in rats. The result suggested that RA exhibits a dose-dependent improvement in high glucose (HG) levels and insulin resistance by reducing the expression of phosphoenolpyruvate carboxykinase (PEPCK) in the liver and enhancing glucose transporter 4 (GLUT4) expression in muscle tissues [34]. Inhibiting α-glucosidase is another highly effective approach to tackle DM as α-glucosidase plays a significant regulatory role in DM [48]. There is evidence to suggest that RA has α-glucosidase inhibitory activity [11,49]. These studies signify the importance of RA in DM treatment; however, ongoing research is focused on further understanding the mechanisms of action of RA in diabetes and its potential applications.

DM is associated with the pathogenesis of various health conditions such as cardiovascular disorders, diabetic nephropathy, diabetic neuropathy (DN), hypertension, and inflammation [50,51]. Various studies have highlighted the role of RA in diabetes-associated secondary health disorders. The role of RA in diabetes-linked nephropathy was studied. HG-stimulated cultured human renal proximal tubular epithelial cells (HK-2) were exposed to RA. RA inhibited connective tissue growth factor (CTGF), which is pathogenic in diabetic nephropathy [52]. Further, in vivo studies were conducted on diabetic rats randomized to receive intragastric doses of RA [52]. RA showed a significant enhancement of renal function and increased body weight in diabetic rats [52]. Peripheral neuropathy is expressed as hypersensitivity to painful stimuli. This complication of diabetes is common in a majority of patients [53].

In a study, rats with DN were studied to understand RA’s role in ameliorating the disease’s pathology. Oral administration of RA elicited antihyperalgesic and antiallodynic effects, attributed to reduced oxidative stress and inflammation associated with DN [54]. According to a study, in diabetic rats, RA application led to notable reductions in glomerular hypertrophy, loss of glomerular count, and glomerulosclerosis [55]. RA is associated with preventing damage related to oxidative stress in the liver and kidneys of diabetic rats [56]. DM is also associated with various cardiovascular disorders. Aortic endothelial function and structure are damaged as a consequence of DM. RA shows protective and anti-inflammatory effects against diabetes-induced damage by restoring vascular endothelial function [12]. Another study reported that RA effectively inhibits lipid peroxidation, consequently preventing the elevation of acetylcholinesterase (AChE) activity in diabetic rats, thereby underscoring the ability of RA to regulate cholinergic neurotransmission and mitigate oxidative damage in the brain during diabetic conditions [57].

### 3.2. RA in Neurodegenerative Diseases

**Figure 3:**
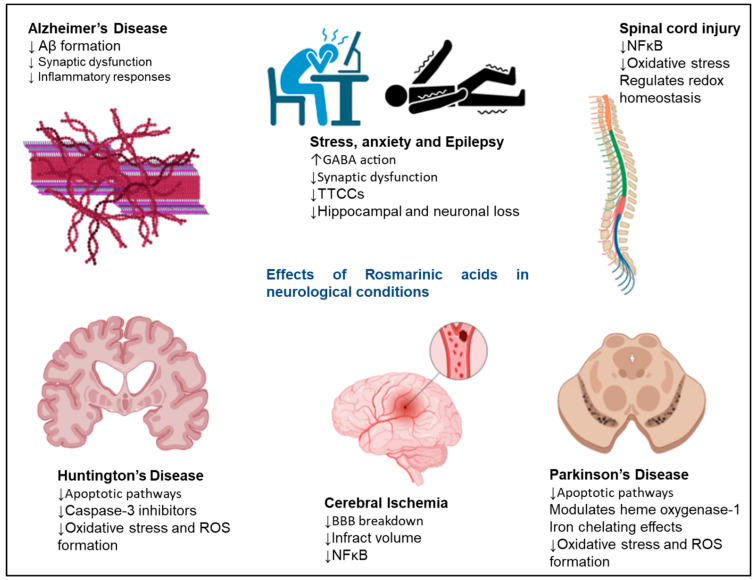
Neuroprotective effects exerted by RA in neurodegenerative disorders. The signalling molecules modulated in the presence of RA to exert neuroprotective effects. RA shows therapeutic effects in AD, Huntington’s disease, cerebral ischaemia, Parkinson’s disease, epilepsy, and stress. The upward arrows represent an increase in function, and the downwards arrows represent a decrease in function of various pathways on RA administration.

Neurodegenerative diseases, or neurodegeneration, arise from the loss or death of neuronal cells in different brain regions. Reports suggest that RA shows neuroprotective effects by reducing oxidative stress and preventing brain cell deaths in vitro against various neurological diseases and neurotoxic molecules [58,59,60,61]. Figure 3 shows the therapeutic roles of RA in various neurological conditions. RA shows neuroprotective effects at the cellular level in various incidences, such as neurodegeneration, chemically induced neurotoxicity, oxidative stress, and neuroinflammation. The neuroprotective effects of RA were evaluated in N2A cells by H_2_O_2_-induced neuronal cell damage; it was observed that the RA treatment suppressed the H_2_O_2_-induced cytotoxicity in N2A cells [38]. The results demonstrated that RA is highly effective in reducing the disturbance of lactate dehydrogenase, preserving mitochondrial membrane potential, and lowering intracellular ROS levels [38]. Overall, the results stressed the fact that RA has the potential to serve as an agent for preventing various human neurodegenerative diseases that result from oxidative stress.

A study investigated the neuroprotective effects of RA on H_2_O_2_-induced neurotoxicity in the human dopaminergic cell line, SH-SY5Y [62]. The results of the study indicated that RA played a significant role in mitigating the production of ROS induced by hydrogen peroxide (H_2_O_2_). RA also showed its effectiveness in suppressing the upregulation of the proapoptotic protein Bax while concurrently down-regulating the levels of the antiapoptotic protein Bcl-2. Additionally, RA exhibited a stimulatory effect on heme oxygenase-1 (HO-1), an antioxidant enzyme. Overall, the results unveiled the potential of RA to protect SH-SY5Y cells when exposed to oxidative stress [62].

#### 3.2.1. Alzheimer’s Disease

The crucial factor in developing Alzheimer’s disease (AD) is oxidative stress, which plays a vital role in free radical production, mitochondria dysfunction, cell death, amyloid beta peptide deposition, and mitochondrial interaction [11]. In an investigation, when amyloid β_(25–35)_-induced AD in rats was treated with RA, which mitigated the impairment of learning and memory disturbance by reducing oxidative stress [63]. Additionally, research on memory impairment explored the potential protective effects of RA using an intracerebroventricular-administered A_(β25–35)_-induced mice model. This study revealed that daily consumption of RA diminished the effect of neurotoxicity of Aβ_25–35_ in mice models by scavenging peroxynitrite (ONOO^−^), preventing memory impairments in AD [64]. RA was evaluated on a cultured neuronal cell line, SH-SY5Y in vitro and ischaemic diabetic stroke in vivo, and the studies revealed that a 50 mg/kg dose of RA decreased high-mobility group box1 (HMGB1) expression, histopathological damage, brain oedema, oxygen-glucose deprivation-induced apoptosis, and cytotoxicity, and blocked TNF-α-induced NF-κB activation in SH-SY5Y cells [65].

#### 3.2.2. Epilepsy

Epilepsy is a central nervous system disorder regarded as a sudden, repetitive seizure because of abnormal behaviour of neuronal cells in the brain—the abnormal behaviour of neuronal cells in the brain results from irregular activities and extreme excitability. Gamma-aminobutyric acid (GABA) is key in maintaining the balance between neuronal excitation and inhibitory tone. Perturbance in this balance is the main factor in promoting epilepsy [66]. RA doses of 100 μg/mL significantly inhibited the activity of gamma-aminobutyric acid transaminase (GABA-T) [67]. Numerous reports showed the protective role of RA in animal models and in vitro studies against epilepsy [68,69]. A study investigated the potential advantages of RA in both in vitro and in vivo models of epileptiform activity induced by pilocarpine. This study revealed RA’s potential to reduce the acute neuromotor disturbances and oxidative damage triggered by status epilepticus (SE) in mice [68]. Furthermore, it appears to offer favourable effects in in vitro models of epileptiform activity, as evidenced by a decrease in lactate release. In another study, two mouse models of acute seizure, 4-aminopyridine (4-AP) and picrotoxin (PTX)-induced seizures were used to investigate the effects of RA. The available drugs, diazepam and valproic acid, were used as positive controls. The outcomes of this study revealed that RA has the capacity to diminish cell damage induced by seizures triggered by 4-AP and PTX, making it a potential candidate for mitigating the pathophysiological processes associated with epilepsy. The outcomes of this study revealed that RA exhibited antioxidant activity, decreased reactive oxygen species production, superoxide dismutase activity, DNA damage, and neuroprotective effects [69].

#### 3.2.3. Huntington’s Disease

Huntington’s disease (HD) is a rare autosomal dominant inherited neurodegenerative disease of the central nervous system due to 36 or more repeats of CAG on the short arm of chromosome 4 in the *Huntingtin* gene. Involuntary choreatic movements, dementia, and psychiatric and behavioural perturbation are the characteristic symptoms of HD [70]. Expression of the *Huntingtin* gene containing CAG repeats results in the production of Huntingtin protein with an expanded polyglutamine region (mutant Huntingtin protein). Mutant Huntingtin protein is related to various synaptic dysfunctions and is directly associated with impaired cellular mechanisms involved in synaptic transmission. Moreover, mutant Huntingtin protein also results in transcriptional dysregulation, glutamate excitotoxicity, and cell death [71]. In an investigation, a rat model of HD, induced by 3-nitro propionic acid (3-Np), was administered RA-loaded solid lipid nanoparticles (SLN) through the nasal passage. The RA-loaded SNL caused resulted in a significant improvement in the behavioural abnormalities and a reduction in oxidative stress. The beneficial outcome of this study led to the conclusion that RA-loaded SLN formulation is an effective therapeutic strategy and can be used as a potential drug in managing HDs [72].

#### 3.2.4. Parkinson’s Disease

Degeneration of dopaminergic neurons in the substantia-nigra of the midbrain, increased iron levels, oxidative stress, and neuronal apoptosis are the main neuropathological features that promote Parkinson’s disease (PD). Thus, any compound that can work against these features of PD can be considered a potential therapeutic molecule [73]. RA effectively mitigates oxidative stress by regulating the neuronal apoptotic process, making it an effective therapeutic agent against PD [62].

The effect of RA was studied on a lesioned rat model of PD induced with 6-OHDA. RA prevented dopamine depletion in the striatum and showed a significant reduction in TH-Positive neurons in the substantia nigra. It also prevented the downregulation of Bcl-2/Bax ratio in 6-OHDA-induced PD rats [59]. Therefore, RA is used in optimizing the mechanical perturbation and disturbances involved in neurodegenerative diseases.

### 3.3. RA in Cancer Therapy

**Figure 4:**
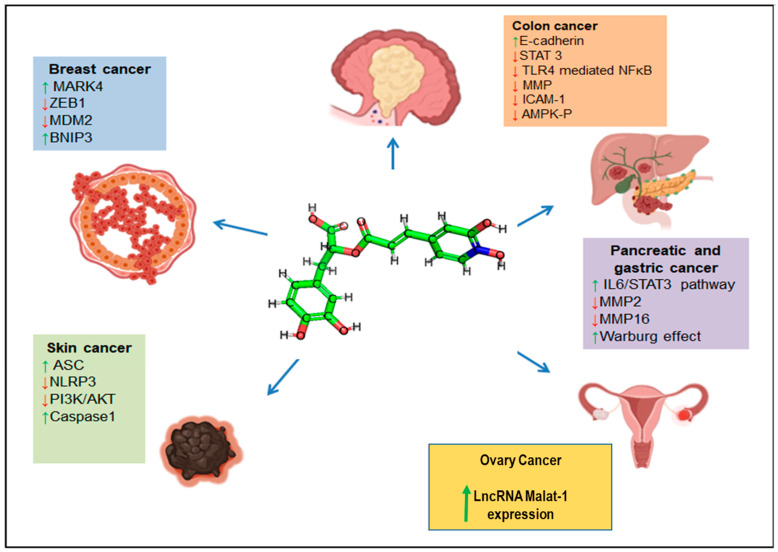
Anticancer effects exerted by RA. Different signalling molecules are modulated in the presence of RA to exert anticancer effects. The blue arrows represent RA’s negative effect on various cancers’ progression. The coloured boxes represent the signalling pathways upregulated (green arrows) or downregulated (red arrows) in the presence of RA.

Cancer is defined by the uncontrollable proliferation of certain cells within the body, which leads to the invasion of adjacent tissues and the dissemination of these cells to distant parts of the body via the lymphatic and circulatory systems, a process known as metastasis [74]. Rosemary and its extracts have been shown to exhibit potential in inhibiting the growth of cancer cells and the development of tumours in various cancer types, including colon, breast, liver, and stomach cancer. In recent times, many studies have reported a wide range of biological properties of RA, thus making it a hit amongst researchers. Globally, pharmaceutical industries are concerned about this natural bioactive compound, and its derivatives as well. Many studies have reported the anticancer potential of RA, thus making it a potential plant-based compound that can be implicated in cancer therapeutics. Figure 4 shows the therapeutic roles of RA in various cancer types.

RA has shown inhibitory roles in various solid tumours by induction of cell cycle arrest, apoptosis, and inhibition of epithelial to mesenchymal transitions and metastasis in a tumour. Studies on the antitumour effects of RA in vitro and in vivo models are shown in Table 2.

#### 3.3.1. Colorectal/Colon Cancers

Metastasis, the dissemination of highly mobile malignant cells, is a phenomenon observed in the majority of cancer patients [89]. Colorectal cancer (CRC) exhibits metastasis in almost 50% of cases and is one of the most commonly diagnosed cancers. Recently, RA has been shown to prevent numerous lethal malignancies, including CRC [90]. A study found that RA inhibited metastasis in the MDA-MB-231B0 and ST-2 breast carcinoma cells [91]. RA treatment in CRC cells inhibited proliferation-induced cell cycle arrest of the G0/G1 phase by reducing the cyclin D1 and CDK4 levels, inhibiting cyclin D1 and CDK4 mRNA expression [79]. In various metastatic phenotypes of CRC cells, RA played a role in regulating epithelial–mesenchymal transition (EMT) through upregulation of an epithelial marker, E-cadherin; and downregulation of mesenchymal markers, such as *N*-cadherin, snail, twist, vimentin, and slug. Treatment with RA inhibited the invasion and migration of CRC cells, and it led to a decrease in the expressions of matrix metalloproteinase (MMP)-2 and MMP-9. All these events resulted from the activation of AMPK [79].

RA showed antitumour effects in colitis-associated colon cancer (CAC) models in in vivo studies [92]. RA suppressed NF-κB and signal transducer and activator of transcription 3 (STAT3) activation in colon cancer cells in an inflammatory microenvironment [92]. Another study documented the anticancer effects in CRC cells by reducing the expression of the proinflammatory gene COX-2; risk factor in tumour development [93]. RA’s influence on tumour metastasis in Ls174-T human colon cancer cells occurred through the modification of the ERK signalling pathway [94].

#### 3.3.2. Breast Cancer

Breast cancer is a medical condition characterized by the uncontrolled growth of abnormal cells within the breast tissue, leading to the formation of tumours. In 2020, there were 2.3 million women diagnosed with breast cancer and 685,000 deaths globally [95]. By the end of 2020, there were 7.8 million women alive who had been diagnosed with breast cancer in the previous 5 years, making it the world’s most prevalent cancer. RA is an effective therapeutic agent against breast cancer and has been documented in various studies [25,91]. RA shows promise as a potential novel treatment strategy for managing bone metastases resulting from breast cancer [91]. This study demonstrated that RA had a dose-dependent inhibitory effect on the migration of MDA-MB-231BO human breast cancer cells, which tend to home in on bone tissue. RA significantly caused dose- and time-dependent cytotoxic and antiproliferative effects in TNBC cell lines of different racial backgrounds, specifically MDA-MB-231 and MDAMB-468 cell lines. RA induced cell cycle arrest-related apoptosis and altered the expression of many apoptosis-involved genes in a different way. In MDA-MB-231 cells, RA halted cell cycle progression in the G0/G1 phase. Conversely, in MDAMB-468 cells, the data indicated that RA resulted in S-phase arrest, leading to a twofold increase in the apoptotic effect compared to MDA-MB-231 cells [25]. This study showed RA to be especially potent against MDA-MB-468 cells in inducing mitotic arrest and apoptosis. Another study reported the inhibition of Microtubule affinity regulating kinase 4 (MARK4) by RA. MARK4 is overexpressed in various cancer types including breast cancer, playing a vital role in the growth, progression, and apoptotic evasion of cancer cells. In this study, treatment with RA induced apoptosis in MDA-MB-231 cells in a dose-dependent manner, highlighting the beneficial effect of RA on breast cancer cells [27].

#### 3.3.3. Non-Small Cell Lung Cancers

RA effectively suppressed the growth of non-small cell lung cancer (NSCLC) cells and induced apoptosis. This apoptotic effect was attained by activating the phosphorylation of JNK (c-Jun *N*-terminal kinase), resulting in a reduction in P-glycoprotein (P-gp) expression, thereby leading to the reversal of P-gp-mediated resistance to cisplatin (DDP) and promoting mitochondria-mediated apoptosis [96]. Another study investigated the effect of rosemary extract (RE) on H1299 human lung cancer cells. The results suggested that treatment with RE dose-dependently inhibited H1299 proliferation with an IC50 value of 19 µg/mL. Further, RE decreased cell survival dose-dependently, and this decrease was associated with elevated levels of cleaved poly (ADP-ribose) polymerase (PARP), which serves as a marker for apoptosis [97].

RA and Cis-diamine dichloro platinum-II (DDP) were studied on A549 and A549DDP cells to study the combinatorial effects on cell viability and apoptosis [98]. After constructing A549DDP, both cell lines were treated with RA, and after 48 h, cell counting kit-8 (CCK8) revealed a reduction in the proliferation rate at variable concentrations. The maximum reduction in proliferation rate was observed at 14.05 µg/mL in A549 cells and 46.47 µg/mL in A549DDP cells. Additionally, co-treatment of RA and DDP showed synergistic effects. Other results demonstrated that RA and DPP treatment for 48 h caused cell cycle arrest at the G1 phase in a concentration-dependent pattern in both NSCLC cell lines.

Further, cell cycle proteins p53 and p21 were examined with co-treatment of RA and DDP. Both proteins increased in the NSCLC cell lines upon RA treatment [94]. Additionally, RA treatment enhanced DDP-induced NSCLC cell apoptosis and the expression of two apoptosis-related proteins, caspase-3 and Bax. RA inhibited the expression of antiapoptotic proteins Bcl-2 and caspase-3 in both A549 and A549DDP cell lines. Further, RA is also involved in upregulating JNK, a member of the MAPK family. c-Jun *N*-terminal kinase inhibitor (JNK), activated through phosphorylation by RA, reduces cell growth and increases apoptosis in NSCLC cell lines. The co-treatment of RA and DDP also inhibits xenograft tumour growth in NSCLC cell lines [96].

#### 3.3.4. Prostate Cancer

RA plays a vital role in prostate cancer therapeutics. A study reported that RA treatment prompted cell cycle arrest and apoptosis in prostate cancer cell lines by influencing the expression of Histone Deacetylase 2 (HDAC2) [99]. In this study, RA exhibited antiprostate cancer (PCa) effects by inhibiting the viability, colony formation, and spheroid formation of PCa cells through the inhibition of HDAC2. This, in turn, led to p53-mediated cell cycle arrest and apoptosis. Another study reported that administration of RE to androgen-insensitive PC-3 prostate cancer cells resulted in notable inhibitions of proliferation, cell survival, and migration, as well as the Akt and mTOR signalling pathways [100].

#### 3.3.5. Gastric Cancer

Gastric cancer is associated with malignancy in the lining of the stomach, which is commonly diagnosed at an advanced stage owing to asymptomatic characteristics at an early stage. It is considered the fourth most common type of cancer and ranks as the second leading cause of cancer-related deaths worldwide [101]. Therefore, it is a great challenge to design and develop a safe, effective, and novel drug for particularly early asymptomatic stages of gastric cancer. A study investigated the effect of RA on some cancer related factors, viz., collagen, MMP, and TIMP, as components of ECM, and on protein glycosylation and MUC1, O-glycosylated oncoprotein in the gastric cancer CRL-1739 cell line. The results of this study revealed the importance of RA in gastric cancer cells. RA reduces the levels of MMP-9 in gastric cancer cells and has a dual effect on TIMP-1 (Tissue Inhibitor of Metalloproteinase-1), i.e., it inhibits TIMP-1 at the protein level but stimulates its expression at the mRNA level. RA stimulates the expression of collagen I and COL1A1 mRNA, while it inhibits the expression of Tn and T antigens, as well as their sialylated forms, in gastric cancer. Moreover, RA inhibits the protein level of MUC1 mucin in gastric cancer [102]. A study showed that RA Analogue-11 induces apoptosis in human gastric cancer SGC-7901 cells through modulation of the EGFR/Akt/NF-κB pathway [84]. MUC1 appears to be a promising target in cancer cells because of its abundant and specifically altered expression and its differential distribution pattern compared to normal tissues. The combination of RA and anti-MUC1 treatment results in greater Gal-3 inhibition compared to the effect of either therapy alone. Gal-3, a β-galactoside binding protein, is a key factor that likely participates in metastasis. Additionally, the combined action of RA and anti-MUC1 significantly induces the expression of the Bax protein and Bad mRNA, which are associated with apoptosis. Furthermore, the mRNA expression of Bcl-2, which is an antiapoptotic protein, is effectively suppressed by the combined action of RA and anti-MUC1 [103].

#### 3.3.6. Cervical Cancer

Cervical cancer ranks as the fourth most common cancer among women globally, with approximately 604,000 new cases reported in 2020. Tragically, of the estimated 342,000 deaths attributed to cervical cancer in the same year, roughly 90% occurred in low- and middle-income countries [104]. Women who are living with HIV are at a significantly higher risk, being approximately six times more likely to develop cervical cancer when compared to women who do not have HIV. Moreover, it is estimated that approximately 5% of all cervical cancer cases can be attributed to HIV infection, highlighting the importance of regular cervical cancer screening and preventative measures for women with HIV to reduce their risk [105].

Cervical cancer is primarily caused by consistent exposure to human papillomavirus (HPV) infection, i.e., more than 95% of cases. The mammalian target of rapamycin (mTOR) and ribosomal protein S6 kinase 1 (S6K1) are the most common signalling pathways that are often activated in cervical cancers [80,106]. mTOR and S6K1 signalling pathways are considered the most appropriate targets against natural compounds for cervical cancer treatment [107]. Cisplatin, in combination with radiotherapy, is used to treat cervical cancers. However, patients with advanced cervical cancer often resist cisplatin, a resistance attributed to the activation of the Akt/mTOR signalling pathway. Numerous studies reported that co-treatment of cisplatin and mTOR inhibitors such as rapamycin cause autophagy and induce apoptosis in cisplatin-resistant cervical cancer cell lines. In a study, it was found that Rosmarinic acid methyl ester (RAME) effectively reduced mTOR-mediated S6K1 activation and the kinase activity of S6K1 by disrupting the interaction between S6K1 and mTOR. The treatment of cervical cancer cells with RAME stimulated autophagy and apoptosis, ultimately resulting in a decrease in the cell survival rate [80].

## 4. Conclusions

This is a comprehensive review on RA, aimed to gather updated research findings, providing a consolidated knowledge base for researchers, clinicians, and policymakers. The present study summarized various mechanisms underlying RA’s therapeutic potential in these diseases. Our review focuses on various aspects of RA’s mechanism of action in these diseases, i.e., it may act by exerting anti-inflammatory and antioxidant effects, inhibiting cell proliferation and migration, and selectively inducing cancer cell apoptosis, exerting antiallergic, antimicrobial, antiviral, and anti-inflammatory activities. Moreover, the present study also emphasized the importance of RA in DM and other secondary complications associated with DM. We have discussed different biological activities of RA and the mechanism of action, focusing on the pathways used in exerting its therapeutic effects. All the research findings suggest that RA is a natural compound with numerous health benefits implicated in different diseases that can be included in the diet with promising effects. In conclusion, this comprehensive review on the biological roles of RA and its implications in diabetes, cancer, and neurodegenerative diseases is not only scientifically significant but also relevant to the broader fields of medicine, biochemistry, and pharmacology. It has the potential to advance our understanding of therapeutic mechanisms and potentially lead to the development of new treatments or preventive strategies.

## 5. Future Prospects

The extensive utilization of RA as an alternative therapy is hindered by its limited bioaccessibility and availability. Additionally, the interaction of RA with various biological components in food contributes to its low bioavailability. RA encounters further absorption challenges, including harsh environmental conditions within the gastrointestinal tract, metabolism within the liver and intestines, and absorption issues. Despite these challenges, RA and its derivatives remain a promising area of research for treating various diseases, such as cancer, diabetes, and neuro-inflammation. Scientists are actively working on improving the bioavailability of RA to mitigate its limitations and reduce side effects. Moreover, a keen interest is in developing new RA derivatives with enhanced biological activity, specificity, and clinical efficacy. Future research should prioritize the development of strategies to enhance the bioavailability of RA. Innovative drug delivery approaches hold the potential for optimizing the therapeutic effects of RA against a spectrum of diseases.

## Figures and Tables

**Table 1:** Biological activity of RA and the mechanism of action and pathways used in exerting the therapeutic effects.

Biological Activity	Biological Activity	References
Anticancer	Inhibits tumour onset and progression Inhibits PI3K/AKT/mTOR signal pathway Decreases the proapoptotic protein expression Decreases leukaemia (HL-60 cells) growth Increases apoptosis in triple-negative breast cancer (TNBC) Decreases hypoxia induce factor HIF-1α levels Inhibits expression of kinases such as MARK4	[22,23,24,25,26,27,28,29]
Cardioprotective	Shows protective effects in myocardial ischaemia by regulating Ca^+2^ homeostasis Inhibits NF-κB signalling pathway Reduces ROS production Has protective effects against lipopolysaccharide-induced cardiac dysfunction	[30,31,32]
Antidiabetic	Increases the expression of key genes associated with mitochondrial biogenesis Increases GLUT4 translocation Reduces the levels of blood glucose, advanced glycation end (AGE) products, cholesterol, lipid peroxides, and triglycerides Inhibits MARK and NF-κB pathways Inhibits proinflammatory T helper and Treg cells Increases diabetes-resistant bacteria	[33,34,35]
Neuroprotective	Shows neuroprotective effects on neurons Restores loss of function in neurons by reducing free radical-mediated oxidative stress Neuroprotective role against oxidative stress in N2A cells Reduces Amyloid-β-induced neurotoxicity by inhibiting oxidative stress	[36,37,38,39]
Antioxidant	Reduces t-BHP-induced oxidative damage Neutralizes free radicals, chelates pro-oxidant ions and reduces lipid peroxidation Inhibits release of IL-6 and ROS Reduces H_2_O_2_-induced cell damage	[40,41,42,43,44,45]

**Table 2:** Therapeutic potential of RA against various cancer cell lines and cancer models, their effective concentrations and effects on cancer cells.

Cancer	Cell Line/Cancer Model	IC_50_/Dosage/Treatment	Effect on Cancer Cell	Ref
Acute lymphoblastic leukaemia	CCRF-CEM CEM/ADR5000 cells	CCRF-CEM and CEM/ADR5000 cells showed IC_50_ of 14.6 μM and 44.5 μM. Treatment for 48 h	Inhibition of IKK-β to block NF-κB signalling. Disruption of MMP and cell adhesion. Stimulated caspase-independent cell death	[75]
Breast cancer	MDA-MB-231 and MDA-MB-468 cells MCF7	MDA-MB-231 and MDA-MB-468 showed IC_50_ 321.75 and 340.45 μM Treatment for 48 h Treatment of 20 and 40 μM RA	RA mediated G0/G1 cell cycle arrest and induction of apoptosis by up-regulating apoptosis-related genes such as *HRK*, *TNFRSF25*, *BNIP3*, *TNF*, *GADD45A*, and downregulating *BIRC5* and *TNFRSF11B* Decreases chemoresistance in breast cancer by regulating methylation patterns via DNMT1	[25,76]
Colorectal cancer (CRC)	HCT8 HCT116 Ls174-T Lovo	IC_50_: 298.1 μM IC_50_: 319.8 μM IC_50_: 539.4 μM IC_50_: 576.3 μM	↓ IL-1β ↓ TNFα ↓ IL-6 ↓ STAT3	[77]
CRC	HCT15	Treatment of RA 10, 50 and 100 μM	Blocks the phospho-ERK pathway and inhibits cell proliferation	[77,78]
CRC	BALB/c mice injected with CT26 via tail vein (lateral)	RA was administered orally (100 mg/kg/day) Duration: 14 days	Apoptosis induction caspases ↑ Bcl-XL ↓ BCL-2 ↓ Induces cell cycle arrest Inhibition of EMT and invasion Reduced metastasis	[79]
Cervical cancer	HeLa and SiHa cells	Combination therapy RA methyl ester and DDP RA 80 µM and DDP 5 µM	Inhibits mTOR/S6K1 pathway to induce apoptosis in cervical cancer	[80]
Glioma	U251 and U343 cells	Treatment with 100, 200, 400 μM RA	Induced apoptosis via ↓ BCL-2 ↑ BAX ↓ caspase-3 ↓ PI3K/AKT/NF-κB	[81]
Glioma	U-87 MG cells	IC_50_: 373.48 μM Treatment time: 48 h	↓ expression of HSP27 ↑ caspase-3	[82]
Gastric cancer	MKN45 cells MKN45 cells injected into BALB/c-nude mice	IC_50_ for 24 h: 240.2 μM Intraperitoneal injection of RA 2 mg/kg for 14 days	Inhibited the signs of the Warburg effect, such as high glucose consumption/anaerobic glycolysis, lactate production/cell acidosis, by inhibiting the IL-6/STAT3 pathway	[83]
Gastric cancer	GES-1 and SGC-7901 cells	IC_50_ 289.425 IC_50_: 73.299 μmol/L Treatment time 24 h	Induced apoptosis via EGFR/AKT/NF-κB pathway	[84]
Hepatocellular carcinoma (HCC)	HepG2 cells	Treatment of 5 and 10 µg/mL of RA	Induced apoptosis via upregulating the mRNA levels of Jun, Jun-B, Fos-B, BAX and caspase-8, and downregulation of mRNA expression of BCL-2	[85]
HCC	H22 tumour-bearing mice	Dosage via intra-peritoneal injection of RA 75, 150, and 300 mg/kg	Inflammatory cytokines ↓ IL-1β ↓ IL-6 ↓ TNF-α ↓ TGF-β ↓ angiogenic factors (VEGF) and phosphorylation of p65	[86]
HCC	SMMC 7721 cells; Tumour-bearing model of nude mice	20, 50, and 100 µmol/L RA treatment; 5, 10, and 20 mg/kg RA administered for 5 days	Inhibition of PI3K/AKT/mTOR pathway resulting in apoptosis, inhibition of EMT in vitro and tumour growth in vivo	[22]
Pancreatic cancer	PANC-1 PATU-8988 MIA PaCa-2 BxPC-3 cells	RA treatment of 100 to 500 μM	Increased degradation of Gli1 Blocks the expression of downstream genes VEGF, Cyclin D1 and snail1 associated with cancer progression	[87]
Pancreatic cells	Tumour-bearing model of nude mice (MIA PaCa-2 cells)	Oral administration of RA 50 mg/kg 30 days	Apoptosis induction and repressed invasion and proliferation of cell Reduction in tumour growth	[87]
Ovarian cancer	SKOV-3 TOV-21G TOV/CisR	Combination therapy RA methyl ester and DDP RA 40 µM and DDP 5 µM	Augmented apoptosis in DDP-resistant ovarian cancer cell line by inhibiting FOXM1	[88]

## Data Availability

All data generated or analysed during this study are included in this manuscript.

## Abbreviations

Rosmarinic acid: RA; 3,4-dihydroxyphenyllactic acid: DHPL; Nuclear Factor Kappa B: NF-κB; Tumour necrosis factor-α: TNF-α; Lipopolysaccharide: LPS; reactive oxygen species: ROS; hydrogen peroxide: H_2_O_2_; inducible nitrogen oxide synthase: iNOS; non-small cell lung cancer: NSCLC; non-Hodgkin lymphomas: NHL; Matrix metalloproteinase: MMP; mitochondrial membrane potential: MMP; transaminase tyrosine aminotransferase: TAT; diabetes mellitus: DM; phosphoenolpyruvate carboxykinase: PEPCK; acetylcholinesterase: AChE; Brain-derived neurotrophic factor: BDNF; Alzheimer’s disease: AD; high-mobility group box1: HMGB1; Gamma-aminobutyric acid transaminase: GABA-T; Huntington’s disease: HD; epithelial–mesenchymal transition: EMT; colorectal cancer: CRC; negative breast cells: TNBC.

## References

[1] Petersen M., Simmonds M.S. (2003). Rosmarinic acid. Phytochemistry.

[2] Scarpati M.L., Oriente G. (1958). Isolamento e costituzione dell’acido rosmarinico (dal *Rosmarinus off.*). Ric. Sci..

[3] Al-Sereiti M., Abu-Amer K., Sena P. (1999). Pharmacology of rosemary (*Rosmarinus officinalis* Linn.) and its therapeutic potentials. Indian J. Exp. Biol..

[4] Zheng W., Wang S.Y. (2001). Antioxidant activity and phenolic compounds in selected herbs. J. Agric. Food Chem..

[5] Takeda H., Tsuji M., Matsumiya T., Kubo M. (2002). Identification of rosmarinic acid as a novel antidepressive substance in the leaves of *Perilla frutescens* Britton var. acuta Kudo (Perillae Herba). Nihon Shinkei Seishin Yakurigaku Zasshi = Jpn. J. Psychopharmacol..

[6] Ito H., Miyazaki T., Ono M., Sakurai H. (1998). Antiallergic activities of rabdosiin and its related compounds: Chemical and biochemical evaluations. Bioorg. Med. Chem..

[7] Wren R., Potter S. (1988). New Cyclopaedia of Botanical Drugs and Preparations.

[8] Luo C., Zou L., Sun H., Peng J., Gao C., Bao L., Ji R., Jin Y., Sun S. (2020). A Review of the Anti-Inflammatory Effects of Rosmarinic Acid on Inflammatory Diseases. Front. Pharmacol..

[9] Hraš A.R., Hadolin M., Knez Ž., Bauman D. (2000). Comparison of antioxidative and synergistic effects of rosemary extract with α-tocopherol, ascorbyl palmitate and citric acid in sunflower oil. Food Chem..

[10] Szabo E., Thelen A., Petersen M. (1999). Fungal elicitor preparations and methyl jasmonate enhance rosmarinic acid accumulation in suspension cultures of *Coleus blumei*. Plant Cell Rep..

[11] Alagawany M., Abd El-Hack M.E., Farag M.R., Gopi M., Karthik K., Malik Y.S., Dhama K. (2017). Rosmarinic acid: Modes of action, medicinal values and health benefits. Anim. Health Res. Rev..

[12] Sotnikova R., Okruhlicova L., Vlkovicova J., Navarova J., Gajdacova B., Pivackova L., Fialova S., Krenek P. (2013). Rosmarinic acid administration attenuates diabetes-induced vascular dysfunction of the rat aorta. J. Pharm. Pharmacol..

[13] Huang N., Hauck C., Yum M.-Y., Rizshsky L., Widrlechner M.P., McCoy J.-A., Murphy P.A., Dixon P.M., Nikolau B.J., Birt D.F. (2009). Rosmarinic acid in *Prunella vulgaris* ethanol extract inhibits lipopolysaccharide-induced prostaglandin E2 and nitric oxide in RAW 264.7 mouse macrophages. J. Agric. Food Chem..

[14] Kim D.-S., Kim H.-R., Woo E.-R., Hong S.-T., Chae H.-J., Chae S.-W. (2005). Inhibitory effects of rosmarinic acid on adriamycin-induced apoptosis in H9c2 cardiac muscle cells by inhibiting reactive oxygen species and the activations of c-Jun N-terminal kinase and extracellular signal-regulated kinase. Biochem. Pharmacol..

[15] Moon D.-O., Kim M.-O., Lee J.-D., Choi Y.H., Kim G.-Y. (2010). Rosmarinic acid sensitizes cell death through suppression of TNF-α-induced NF-κB activation and ROS generation in human leukemia U937 cells. Cancer Lett..

[16] Osakabe N., Yasuda A., Natsume M., Yoshikawa T. (2004). Rosmarinic acid inhibits epidermal inflammatory responses: Anticarcinogenic effect of *Perilla frutescens* extract in the murine two-stage skin model. Carcinogenesis.

[17] Ramos-Hryb A.B., Cunha M.P., Kaster M.P., Rodrigues A.L.S., Attaur R. (2018). Chapter 6—Natural Polyphenols and Terpenoids for Depression Treatment: Current Status. Studies in Natural Products Chemistry.

[18] Oh H.-A., Park C.-S., Ahn H.-J., Park Y.S., Kim H.-M. (2011). Effect of Perilla frutescens var. acuta Kudo and rosmarinic acid on allergic inflammatory reactions. Exp. Biol. Med..

[19] Bulgakov V.P., Inyushkina Y.V., Fedoreyev S.A. (2012). Rosmarinic acid and its derivatives: Biotechnology and applications. Crit. Rev. Biotechnol..

[20] Karthikkumar V., Sivagami G., Vinothkumar R., Rajkumar D., Nalini N. (2012). Modulatory efficacy of rosmarinic acid on premalignant lesions and antioxidant status in 1,2-dimethylhydrazine induced rat colon carcinogenesis. Environ. Toxicol. Pharmacol..

[21] Amoah S.K., Sandjo L.P., Kratz J.M., Biavatti M.W. (2016). Rosmarinic acid–pharmaceutical and clinical aspects. Planta Medica.

[22] Wang L., Yang H., Wang C., Shi X., Li K. (2019). Rosmarinic acid inhibits proliferation and invasion of hepatocellular carcinoma cells SMMC 7721 via PI3K/AKT/mTOR signal pathway. Biomed. Pharmacother..

[23] Zhao J., Xu L., Jin D., Xin Y., Tian L., Wang T., Zhao D., Wang Z., Wang J. (2022). Rosmarinic acid and related dietary supplements: Potential applications in the prevention and treatment of cancer. Biomolecules.

[24] Saiko P., Steinmann M.-T., Schuster H., Graser G., Bressler S., Giessrigl B., Lackner A., Grusch M., Krupitza G., Bago-Horvath Z. (2015). Epigallocatechin gallate, ellagic acid, and rosmarinic acid perturb dNTP pools and inhibit de novo DNA synthesis and proliferation of human HL-60 promyelocytic leukemia cells: Synergism with arabinofuranosylcytosine. Phytomedicine.

[25] Messeha S.S., Zarmouh N.O., Asiri A., Soliman K.F. (2020). Rosmarinic acid-induced apoptosis and cell cycle arrest in triple-negative breast cancer cells. Eur. J. Pharmacol..

[26] Jeon Y.J., Song K.S., Han H.J., Park S.H., Chang W., Lee M.Y. (2014). Rosmarinic acid inhibits chemical hypoxia-induced cytotoxicity in primary cultured rat hepatocytes. Arch. Pharmacal Res..

[27] Anwar S., Shamsi A., Shahbaaz M., Queen A., Khan P., Hasan G.M., Islam A., Alajmi M.F., Hussain A., Ahmad F. (2020). Rosmarinic acid exhibits anticancer effects via MARK4 inhibition. Sci. Rep..

[28] Noor S., Mohammad T., Rub M.A., Raza A., Azum N., Yadav D.K., Hassan M.I., Asiri A.M. (2022). Biomedical features and therapeutic potential of rosmarinic acid. Arch. Pharmacal Res..

[29] Sadeghi A., Bastin A.R., Ghahremani H., Doustimotlagh A.H. (2020). The effects of rosmarinic acid on oxidative stress parameters and inflammatory cytokines in lipopolysaccharide-induced peripheral blood mononuclear cells. Mol. Biol. Rep..

[30] Javidanpour S., Dianat M., Badavi M., Mard S.A. (2017). The cardioprotective effect of rosmarinic acid on acute myocardial infarction and genes involved in Ca^2+^ homeostasis. Free Radic. Res..

[31] Quan W., Liu H., Zhang W., Lou W., Gong Y., Yuan C., Shao Q., Wang N., Guo C., Liu F. (2021). Cardioprotective effect of rosmarinic acid against myocardial ischaemia/reperfusion injury via suppression of the NF-κB inflammatory signalling pathway and ROS production in mice. Pharm. Biol..

[32] Peng K., Yang F., Qiu C., Yang Y., Lan C. (2023). Rosmarinic acid protects against lipopolysaccharide-induced cardiac dysfunction via activating Sirt1/PGC-1α pathway to alleviate mitochondrial impairment. Clin. Exp. Pharmacol. Physiol..

[33] Ngo Y.L., Lau C.H., Chua L.S. (2018). Review on rosmarinic acid extraction, fractionation and its anti-diabetic potential. Food Chem. Toxicol..

[34] Runtuwene J., Cheng K.-C., Asakawa A., Amitani H., Amitani M., Morinaga A., Takimoto Y., Kairupan B.H.R., Inui A. (2016). Rosmarinic acid ameliorates hyperglycemia and insulin sensitivity in diabetic rats, potentially by modulating the expression of PEPCK and GLUT4. Drug Des. Dev. Ther..

[35] Nyandwi J.B., Ko Y.S., Jin H., Yun S.P., Park S.W., Kim H.J. (2021). Rosmarinic acid exhibits a lipid-lowering effect by modulating the expression of reverse cholesterol transporters and lipid metabolism in high-fat diet-fed mice. Biomolecules.

[36] Braidy N., Matin A., Rossi F., Chinain M., Laurent D., Guillemin G. (2014). Neuroprotective effects of rosmarinic acid on ciguatoxin in primary human neurons. Neurotox. Res..

[37] Shang A.-J., Yang Y., Wang H.-Y., Tao B.-Z., Wang J., Wang Z.-F., Zhou D.-B. (2017). Spinal cord injury effectively ameliorated by neuroprotective effects of rosmarinic acid. Nutr. Neurosci..

[38] Ghaffari H., Venkataramana M., Ghassam B.J., Nayaka S.C., Nataraju A., Geetha N., Prakash H. (2014). Rosmarinic acid mediated neuroprotective effects against H2O2-induced neuronal cell damage in N2A cells. Life Sci..

[39] Iuvone T., De Filippis D., Esposito G., D’Amico A., Izzo A.A. (2006). The spice sage and its active ingredient rosmarinic acid protect PC12 cells from amyloid-β peptide-induced neurotoxicity. J. Pharmacol. Exp. Ther..

[40] Lima C.F., Valentao P.C., Andrade P.B., Seabra R.M., Fernandes-Ferreira M., Pereira-Wilson C. (2007). Water and methanolic extracts of Salvia officinalis protect HepG2 cells from t-BHP induced oxidative damage. Chem. Biol. Interact..

[41] Ferraro V., Madureira A.R., Sarmento B., Gomes A., Pintado M.E. (2015). Study of the interactions between rosmarinic acid and bovine milk whey protein α-Lactalbumin, β-Lactoglobulin and Lactoferrin. Food Res. Int..

[42] Li G.-S., Jiang W.-L., Tian J.-W., Qu G.-W., Zhu H.-B., Fu F.-H. (2010). In vitro and in vivo antifibrotic effects of rosmarinic acid on experimental liver fibrosis. Phytomedicine.

[43] Li P., Yang X., Lee W.J., Huang F., Wang Y., Li Y. (2021). Comparison between synthetic and rosemary-based antioxidants for the deep frying of French fries in refined soybean oils evaluated by chemical and non-destructive rapid methods. Food Chem..

[44] Vostálová J., Zdařilová A., Svobodová A. (2010). Prunella vulgaris extract and rosmarinic acid prevent UVB-induced DNA damage and oxidative stress in HaCaT keratinocytes. Arch. Dermatol. Res..

[45] Gao L., Wei H., Zhao H., Xiao S., Zheng R. (2005). Antiapoptotic and antioxidant effects of rosmarinic acid in astrocytes. Die Pharm. Int. J. Pharm. Sci..

[46] Bao T.-Q., Li Y., Qu C., Zheng Z.-G., Yang H., Li P. (2020). Antidiabetic effects and mechanisms of rosemary (*Rosmarinus officinalis* L.) and its phenolic components. Am. J. Chin. Med..

[47] Wilcox G. (2005). Insulin and insulin resistance. Clin. Biochem. Rev..

[48] Liu S.-K., Hao H., Bian Y., Ge Y.-X., Lu S., Xie H.-X., Wang K.-M., Tao H., Yuan C., Zhang J. (2021). Discovery of new α-glucosidase inhibitors: Structure-based virtual screening and biological evaluation. Front. Chem..

[49] Zhu F., Asada T., Sato A., Koi Y., Nishiwaki H., Tamura H. (2014). Rosmarinic acid extract for antioxidant, antiallergic, and α-glucosidase inhibitory activities, isolated by supramolecular technique and solvent extraction from Perilla leaves. J. Agric. Food Chem..

[50] Koye D.N., Magliano D.J., Nelson R.G., Pavkov M.E. (2018). The global epidemiology of diabetes and kidney disease. Adv. Chronic Kidney Dis..

[51] Lee H.J., Seo H.I., Cha H.Y., Yang Y.J., Kwon S.H., Yang S.J. (2018). Diabetes and Alzheimer’s disease: Mechanisms and nutritional aspects. Clin. Nutr. Res..

[52] Jiang W.L., Xu Y., Zhang S.P., Hou J., Zhu H.B. (2012). Effect of rosmarinic acid on experimental diabetic nephropathy. Basic Clin. Pharmacol. Toxicol..

[53] Feldman E.L., Callaghan B.C., Pop-Busui R., Zochodne D.W., Wright D.E., Bennett D.L., Bril V., Russell J.W., Viswanathan V. (2019). Diabetic neuropathy. Nat. Rev. Dis. Primers.

[54] Hasanein P., Mohammad Zaheri L. (2014). Effects of rosmarinic acid on an experimental model of painful diabetic neuropathy in rats. Pharm. Biol..

[55] Tavafi M., Ahmadvand H., Tamjidipoor A. (2011). Rosmarinic acid ameliorates diabetic nephropathy in uninephrectomized diabetic rats. Iran. J. Basic Med. Sci..

[56] Mushtaq N., Schmatz R., Ahmed M., Pereira L.B., da Costa P., Reichert K.P., Dalenogare D., Pelinson L.P., Vieira J.M., Stefanello N. (2015). Protective effect of rosmarinic acid against oxidative stress biomarkers in liver and kidney of strepotozotocin-induced diabetic rats. J. Physiol. Biochem..

[57] Mushtaq N., Schmatz R., Pereira L.B., Ahmad M., Stefanello N., Vieira J.M., Abdalla F., Rodrigues M.V., Baldissarelli J., Pelinson L.P. (2014). Rosmarinic acid prevents lipid peroxidation and increase in acetylcholinesterase activity in brain of streptozotocin-induced diabetic rats. Cell Biochem. Funct..

[58] Sepand M.R., Soodi M., Hajimehdipoor H., Soleimani M., Sahraei E. (2013). Comparison of neuroprotective effects of *Melissa officinalis* total extract and its acidic and non-acidic fractions against a β-induced toxicity. Iran. J. Pharm. Res. IJPR.

[59] Wang J., Xu H., Jiang H., Du X., Sun P., Xie J. (2012). Neurorescue effect of rosmarinic acid on 6-hydroxydopamine-lesioned nigral dopamine neurons in rat model of Parkinson’s disease. J. Mol. Neurosci..

[60] Choi H.R., Choi J.S., Han Y.N., Bae S.J., Chung H.Y. (2002). Peroxynitrite scavenging activity of herb extracts. Phytother. Res..

[61] Qiao S., Li W., Tsubouchi R., Haneda M., Murakami K., Takeuchi F., Nisimoto Y., Yoshino M. (2005). Rosmarinic acid inhibits the formation of reactive oxygen and nitrogen species in RAW264. 7 macrophages. Free Radic. Res..

[62] Lee H.J., Cho H.-S., Park E., Kim S., Lee S.-Y., Kim C.-S., Kim D.K., Kim S.-J., Chun H.S. (2008). Rosmarinic acid protects human dopaminergic neuronal cells against hydrogen peroxide-induced apoptosis. Toxicology.

[63] Baluchnejadmojarad T., Roghani M., Kazemloo P. (2013). Rosmarinic acid mitigates learning and memory disturbances in amyloid β (25–35)-induced model of Alzheimer’s disease in rat. J. Basic Clin. Pathophysiol..

[64] Alkam T., Nitta A., Mizoguchi H., Itoh A., Nabeshima T. (2007). A natural scavenger of peroxynitrites, rosmarinic acid, protects against impairment of memory induced by Aβ25–35. Behav. Brain Res..

[65] Luan H., Kan Z., Xu Y., Lv C., Jiang W. (2013). Rosmarinic acid protects against experimental diabetes with cerebral ischemia: Relation to inflammation response. J. Neuroinflamm..

[66] Treiman D.M. (2001). GABAergic mechanisms in epilepsy. Epilepsia.

[67] Awad R., Muhammad A., Durst T., Trudeau V.L., Arnason J.T. (2009). Bioassay-guided fractionation of lemon balm (*Melissa officinalis* L.) using an in vitro measure of GABA transaminase activity. Phytother. Res. Int. J. Devoted Pharmacol. Toxicol. Eval. Nat. Prod. Deriv..

[68] Neuberger B., Mello F.K., Mallmann M.P., da Costa Sobral K.G., Fighera M.R., Royes L.F.F., Furian A.F., Sampaio T.B., Oliveira M.S. (2023). Beneficial Effects of Rosmarinic Acid In vitro and In vivo Models of Epileptiform Activity Induced by Pilocarpine. Brain Sci..

[69] Luft J.G., Steffens L., Morás A.M., da Rosa M.S., Leipnitz G., Regner G.G., Pflüger P.F., Gonçalves D., Moura D.J., Pereira P. (2019). Rosmarinic acid improves oxidative stress parameters and mitochondrial respiratory chain activity following 4-aminopyridine and picrotoxin-induced seizure in mice. Naunyn-Schmiedeberg’s Arch. Pharmacol..

[70] Roos R.A. (2010). Huntington’s disease: A clinical review. Orphanet J. Rare Dis..

[71] Li J.-Y., Plomann M., Brundin P. (2003). Huntington’s disease: A synaptopathy?. Trends Mol. Med..

[72] Bhatt R., Singh D., Prakash A., Mishra N. (2015). Development, characterization and nasal delivery of rosmarinic acid-loaded solid lipid nanoparticles for the effective management of Huntington’s disease. Drug Deliv..

[73] Ghasemzadeh Rahbardar M., Hosseinzadeh H. (2020). Effects of rosmarinic acid on nervous system disorders: An updated review. Naunyn-Schmiedeberg’s Arch. Pharmacol..

[74] Buga A.-M., Docea A.O., Albu C., Malin R.D., Branisteanu D.E., Ianosi G., Ianosi S.L., Iordache A., Calina D. (2019). Molecular and cellular stratagem of brain metastases associated with melanoma. Oncol. Lett..

[75] Wu C.-F., Hong C., Klauck S.M., Lin Y.-L., Efferth T. (2015). Molecular mechanisms of rosmarinic acid from *Salvia miltiorrhiza* in acute lymphoblastic leukemia cells. J. Ethnopharmacol..

[76] Paluszczak J., Krajka-Kuźniak V., Baer-Dubowska W. (2010). The effect of dietary polyphenols on the epigenetic regulation of gene expression in MCF7 breast cancer cells. Toxicol. Lett..

[77] Xu Y., Han S., Lei K., Chang X., Wang K., Li Z., Liu J. (2016). Anti-Warburg effect of rosmarinic acid via miR-155 in colorectal carcinoma cells. Eur. J. Cancer Prev..

[78] Xavier C.P., Lima C.F., Fernandes-Ferreira M., Pereira-Wilson C. (2009). *Salvia fruticosa*, *Salvia officinalis*, and rosmarinic acid induce apoptosis and inhibit proliferation of human colorectal cell lines: The role in MAPK/ERK pathway. Nutr. Cancer.

[79] Han Y.-H., Kee J.-Y., Hong S.-H. (2018). Rosmarinic acid activates AMPK to inhibit metastasis of colorectal cancer. Front. Pharmacol..

[80] Nam K.H., Yi S.A., Nam G., Noh J.S., Park J.W., Lee M.G., Park J.H., Oh H., Lee J., Lee K.R. (2019). Identification of a novel S6K1 inhibitor, rosmarinic acid methyl ester, for treating cisplatin-resistant cervical cancer. BMC Cancer.

[81] Liu Y., Xu X., Tang H., Pan Y., Hu B., Huang G. (2021). Rosmarinic acid inhibits cell proliferation, migration, and invasion and induces apoptosis in human glioma cells. Int. J. Mol. Med..

[82] Şengelen A., Önay-Uçar E. (2018). Rosmarinic acid and siRNA combined therapy represses Hsp27 (HSPB1) expression and induces apoptosis in human glioma cells. Cell Stress Chaperones.

[83] Han S., Yang S., Cai Z., Pan D., Li Z., Huang Z., Zhang P., Zhu H., Lei L., Wang W. (2015). Anti-Warburg effect of rosmarinic acid via miR-155 in gastric cancer cells. Drug Des. Dev. Ther..

[84] Li W., Li Q., Wei L., Pan X., Huang D., Gan J., Tang S. (2019). Rosmarinic acid analogue-11 induces apoptosis of human gastric cancer SGC-7901 cells via the epidermal growth factor receptor (EGFR)/Akt/nuclear factor kappa B (NF-κB) pathway. Med. Sci. Monit. Basic Res..

[85] Lin C.-S., Kuo C.-L., Wang J.-P., Cheng J.-S., Huang Z.-W., Chen C.-F. (2007). Growth inhibitory and apoptosis inducing effect of *Perilla frutescens* extract on human hepatoma HepG2 cells. J. Ethnopharmacol..

[86] Cao W., Hu C., Wu L., Xu L., Jiang W. (2016). Rosmarinic acid inhibits inflammation and angiogenesis of hepatocellular carcinoma by suppression of NF-κB signaling in H22 tumor-bearing mice. J. Pharmacol. Sci..

[87] Zhou X., Wang W., Li Z., Chen L., Wen C., Ruan Q., Xu Z., Liu R., Xu J., Bai Y. (2022). Rosmarinic acid decreases the malignancy of pancreatic cancer through inhibiting Gli1 signaling. Phytomedicine.

[88] Lim S.H., Nam K.H., Kim K., Yi S.A., Lee J., Han J.-W. (2020). Rosmarinic acid methyl ester regulates ovarian cancer cell migration and reverses cisplatin resistance by inhibiting the expression of Forkhead Box M1. Pharmaceuticals.

[89] Dhyani P., Quispe C., Sharma E., Bahukhandi A., Sati P., Attri D.C., Szopa A., Sharifi-Rad J., Docea A.O., Mardare I. (2022). Anticancer potential of alkaloids: A key emphasis to colchicine, vinblastine, vincristine, vindesine, vinorelbine and vincamine. Cancer Cell Int..

[90] Wang C.-Z., Zhang Z., Anderson S., Yuan C.-S. (2014). Natural products and chemotherapeutic agents on cancer: Prevention vs. treatment. Am. J. Chin. Med..

[91] Xu Y., Jiang Z., Ji G., Liu J. (2010). Inhibition of bone metastasis from breast carcinoma by rosmarinic acid. Planta Medica.

[92] Jin B.-R., Chung K.-S., Hwang S., Hwang S.N., Rhee K.-J., Lee M., An H.-J. (2021). Rosmarinic acid represses colitis-associated colon cancer: A pivotal involvement of the TLR4-mediated NF-κB-STAT3 axis. Neoplasia.

[93] Scheckel K.A., Degner S.C., Romagnolo D.F. (2008). Rosmarinic acid antagonizes activator protein-1–dependent activation of cyclooxygenase-2 expression in human cancer and nonmalignant cell lines. J. Nutr..

[94] Chaitanya M.V.N.L., Ramanunny A.K., Babu M.R., Gulati M., Vishwas S., Singh T.G., Chellappan D.K., Adams J., Dua K., Singh S.K. (2022). Journey of Rosmarinic acid as biomedicine to nano-biomedicine for treating cancer: Current strategies and future perspectives. Pharmaceutics.

[95] Banmare S., Mude G. (2023). Awareness regarding breast cancer among the female population in Wardha District. F1000Research.

[96] Liao X.Z., Gao Y., Sun L.L., Liu J.H., Chen H.R., Yu L., Chen Z.Z., Chen W.H., Lin L.Z. (2020). Rosmarinic acid reverses non-small cell lung cancer cisplatin resistance by activating the MAPK signaling pathway. Phytother. Res..

[97] O’Neill E.J., Moore J., Song J., Tsiani E.L. (2021). Inhibition of non-small cell lung cancer proliferation and survival by rosemary extract is associated with activation of ERK and AMPK. Life.

[98] Chen Y., Zhang Y., Song W., Zhang Y., Dong X., Tan M. (2020). Ginsenoside Rh2 improves the cisplatin anti-tumor effect in lung adenocarcinoma A549 cells via superoxide and PD-L1. Anti-Cancer Agents Med. Chem..

[99] Jang Y.-G., Hwang K.-A., Choi K.-C. (2018). Rosmarinic acid, a component of rosemary tea, induced the cell cycle arrest and apoptosis through modulation of HDAC2 expression in prostate cancer cell lines. Nutrients.

[100] Jaglanian A., Termini D., Tsiani E. (2020). Rosemary (*Rosmarinus officinalis* L.) extract inhibits prostate cancer cell proliferation and survival by targeting Akt and mTOR. Biomed. Pharmacother..

[101] Qiu M., Xu R. (2013). The progress of targeted therapy in advanced gastric cancer. Biomark. Res..

[102] Radziejewska I., Supruniuk K., Nazaruk J., Karna E., Popławska B., Bielawska A., Galicka A. (2018). Rosmarinic acid influences collagen, MMPs, TIMPs, glycosylation and MUC1 in CRL-1739 gastric cancer cell line. Biomed. Pharmacother..

[103] Radziejewska I., Supruniuk K., Bielawska A. (2021). Anti-cancer effect of combined action of anti-MUC1 and rosmarinic acid in AGS gastric cancer cells. Eur. J. Pharmacol..

[104] Sung H., Ferlay J., Siegel R.L., Laversanne M., Soerjomataram I., Jemal A., Bray F. (2021). Global cancer statistics 2020: GLOBOCAN estimates of incidence and mortality worldwide for 36 cancers in 185 countries. CA Cancer J. Clin..

[105] Stelzle D., Tanaka L.F., Lee K.K., Khalil A.I., Baussano I., Shah A.S., McAllister D.A., Gottlieb S.L., Klug S.J., Winkler A.S. (2021). Estimates of the global burden of cervical cancer associated with HIV. Lancet Glob. Health.

[106] Selman C., Tullet J.M., Wieser D., Irvine E., Lingard S.J., Choudhury A.I., Claret M., Al-Qassab H., Carmignac D., Ramadani F. (2009). Ribosomal protein S6 kinase 1 signaling regulates mammalian life span. Science.

[107] Jeon Y.J., Yi S.A., Lee J., Han J.-W. (2022). Nuclear S6K1 regulates cAMP-responsive element-dependent gene transcription through activation of mTOR signal pathway. Biochem. Biophys. Res. Commun..

